# Efficacy of Steroid Pulse Therapy for Autoimmune Pancreatitis Type 1: A Retrospective Study

**DOI:** 10.1371/journal.pone.0138604

**Published:** 2015-09-18

**Authors:** Mitsuru Sugimoto, Tadayuki Takagi, Rei Suzuki, Naoki Konno, Ko Watanabe, Jun Nakamura, Hitomi Kikuchi, Yuichi Waragai, Hiroyuki Asama, Mika Takasumi, Takuto Hikichi, Hiroshi Watanabe, Katsutoshi Obara, Hiromasa Ohira

**Affiliations:** 1 Department of Gastroenterology and Rheumatology, Fukushima Medical University, School of Medicine, Fukushima, Japan; 2 Department of Endoscopy, Fukushima Medical University Hospital, Fukushima, Japan; RWTH Aachen, GERMANY

## Abstract

Autoimmune pancreatitis (AIP) is treatable with steroids, but relapse is frequent. The efficacy of steroid pulse therapy has been shown for various autoimmune diseases, but has not become established therapy. In this study, we reviewed the efficacy of steroid pulse therapy in 24 subjects who were diagnosed with AIP type 1 at our hospital. Patient characteristics, time-course of serum IgG4, and the cumulative relapse-free survival rate were compared between patients who received oral steroid therapy (oral group) and those who were treated with steroid pulse therapy (pulse group). Serum IgG4 was reduced significantly after therapy in both groups and the 5-year cumulative relapse-free survival rates in the two groups did not differ significantly (oral group 46.9%, pulse group 77.8%). However, in a subset of cases with diffuse pancreatic swelling, this rate was significantly lower in the oral group (33.3% vs. 100.0%, *p* = 0.046). These results suggest that steroid pulse therapy is effective for prevention of relapse in AIP patients with diffuse pancreatic swelling.

## Introduction

Autoimmune pancreatitis (AIP) was defined by Yoshida et al. as pancreatitis caused by irregular narrowing of the pancreatic duct, pancreatic swelling, or infiltration and fibrillation of lymphocytes, with such events related to autoimmune mechanisms [1]. Hamano et al. reported rising levels of serum IgG4 in patients with AIP [2]. The 2010 International Consensus Diagnostic Criteria (ICDC) for Autoimmune Pancreatitis [3] define pancreatitis as “Type 1” when other organ involvement and elevated serum IgG4 are present and lymphoplasmacytic sclerosing pancreatitis (LPSP) is histologically the distinguishing characteristic; or “Type 2” when elevated serum IgG4 is not present, the symptoms accompany inflammatory bowel disease, and idiopathic duct-centric chronic pancreatitis (IDCP) / granulocytic epithelial lesion (GEL) are histologically the distinguishing characteristics. Due to growing recognition of AIP, the number of reported cases has increased. Steroid therapy is the standard treatment, but relapse is reported to occur in 10–53% of cases [4–7].

Steroid pulse therapy produces local immunosuppression after organ transplantation and is effective for systemic lupus erythematosus (SLE), intestinal pneumonia, and several autoimmune diseases [8–10], but has not become established therapy for AIP. Matsushita et al. suggested that steroid pulse therapy can be used for lower bile duct stricture and is useful for following the therapeutic outcome because of the lack of a need for tapering [11]. In 2011, Tomiyama et al. reported significant improvements of γ-guanosine triphosphate (GTP) at 2 weeks after steroid pulse therapy, alanine aminotransferase (ALT) at 2 and 8 weeks, and glycosylated hemoglobin at 7 months after therapy [12]. In addition, γ-GTP at 2 and 8 weeks after therapy were improved in a subset of patients with diffuse pancreatic swelling.

Oral steroid therapy with prednisolone (PSL) and steroid pulse therapy with methylprednisolone (mPSL) are administered for AIP in our hospital. The goal of this study was to retrospectively review the efficacy of steroid pulse therapy for AIP.

## Patients and Methods

### Patients

**Fig 1:**
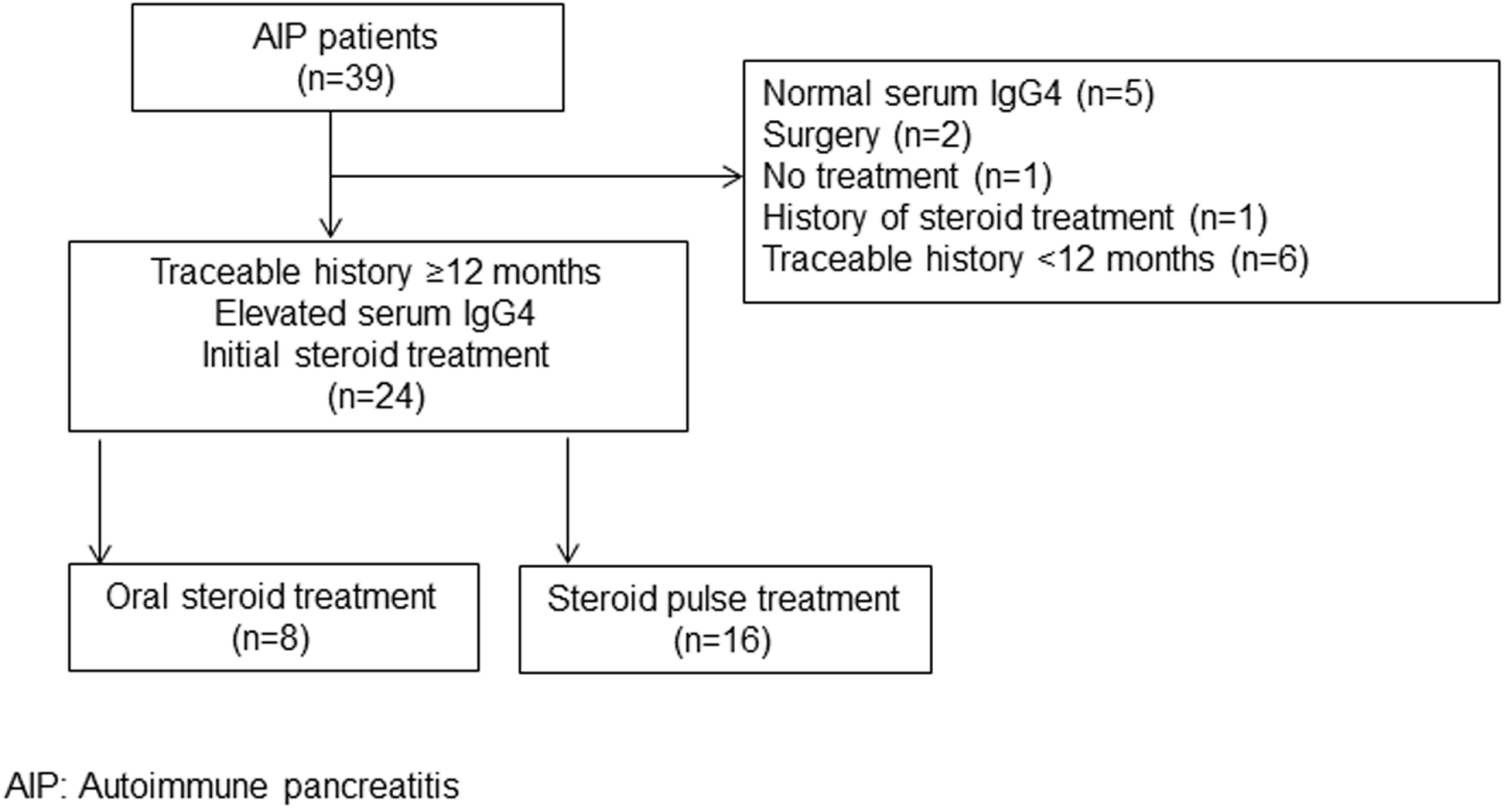
Breakdown of AIP patients in our hospital.

This study was approved by the Ethics Committee of Fukushima Medical University Hospital. Patients were not required to give informed consent to the study because the analysis uses anonymous clinical data obtained after each patient agreed to treatment by written consent. For full disclosure, the details of the study are published on the home page of Fukushima Medical University. Among 39 patients with AIP treated at our hospital from July 2003 to July 2013, 24 AIP type 1 patients with traceable treatment histories of ≥12 months and initial elevated serum IgG4 ≥135 mg/dl who were initially treated with steroid therapy were selected for the study (Fig 1). Patients with normal serum IgG4 (n = 5), surgery as initial treatment (n = 2), no treatment (n = 1), history of steroid treatment (n = 1), or a traceable history < 12 months (n = 6) were excluded from the study. The normal range of serum IgG4 is 4.8–105 mg/dl in our hospital.

All patients were classified as “unconfirmed diagnosis” or higher based on the 2010 International Consensus Diagnostic Criteria for Autoimmune Pancreatitis (confirmed diagnosis: 22 cases, quasi-confirmed diagnosis: 2 cases). All underwent endoscopic ultrasonograpy-guided fine needle aspiration (EUS-FNA) for the purpose of cytology to rule out pancreatic cancer, therefore, there were no diagnoses of LPSP based on EUS-FNA specimens.

### Therapy

Treatment regimens included (1) starting with peroral PSL 30 mg, reducing the dose in 5-mg increments every 4 weeks down to 10 mg, and then reducing the dose in 2.5-mg increments (8 patients, oral group); and (2) intravenous administration of mPSL 250 mg/day or 125 mg/day for 3 days, followed by starting peroral PSL 20 mg and reducing the dose as in method (1) (16 patients, pulse group: steroid pulse therapy with mPSL 250 mg/day in 11 patients, and with mPSL 125 mg/day in 5 patients). There were no specific conditions for steroid pulse therapy and treatment regimens were selected randomly by each attending physician.

### Definition of relapse

Patients were diagnosed with relapse after recovery following steroid treatment based on observation of pancreatitis or cholangitis with re-elevated serum IgG4 (≥135 mg/dl). Patients were diagnosed with newly-developed cholangitis after pancreatitis if focal or multiple stenoses of extrahepatic or intrahepatic bile ducts were detected by endoscopic retrograde cholangiopancreatography.

### Examination items

The oral and pulse groups were compared based on age, gender, type of pancreatic swelling (diffuse or focal), serum IgG4 before treatment, other organ involvement, obstructive jaundice, and PSL dosage (dosage of mPSL was converted to PSL) until serum IgG4 reached a minimum level, and the number of patients with elevated glycosylated hemoglobin (HbA1c) after steroid therapy. To determine the impact of steroid treatment on the long-term prognosis, serum IgG4 levels were compared before and after treatment. Serum IgG4 after therapy was defined as the minimum value before relapse in patients with relapse. The cumulative relapse-free survival rate was compared between the two groups and was also examined in a subset of patients who showed diffuse pancreatic swelling. As mentioned above, Tomiyama et al. found that γ-GTP at 8 weeks after therapy was improved by steroid pulse therapy only in patients with diffuse pancreatic swelling [12]. Thus, we thought that steroid pulse therapy was more likely to be effective in AIP patients with diffuse pancreatic swelling. Therefore, the cumulative relapse-free survival rate was also compared in patients with diffuse pancreatic swelling (3 in the oral group and 6 in the pulse group).

### Statistics

Age, PSL dosage until serum IgG4 reached a minimum level, and serum IgG4 levels were compared by Mann-Whitney U test. Comparison of serum IgG4 levels before and after treatment in each group were compared by Wilcoxon signed-rank test. Gender, locality of pancreatic enlargement (diffuse or focal), other organ involvement, and the presence of obstructive jaundice, and the number of patients with elevated HbA1c after steroid therapy were compared by Fisher exact probability test. A Log-rank test was used for comparison of cumulative relapse-free survival rates. *P* < 0.05 was considered to be significant in all tests. All analyses were performed using Statcel 3 (OMS Edition, Saitama, Japan).

## Results

There were no significant differences between the oral and pulse groups for age (56.0±9.4 vs. 60.0±9.6, *P* = 0.41), gender (men 8, women 0 vs. men 14, women 2, *P* = 0.43), type of pancreatic swelling (diffuse 3, partial 5 vs. diffuse 5, partial 11, *P* = 0.55), serum IgG4 before treatment (367.5±287.2 vs. 476.0±409.3 mg/dl, *P* = 0.27), other organ involvement (4 vs. 9, *P* = 0.56), involvement of obstructive jaundice (4 vs. 8, *P* = 0.67), and PSL dosage until serum IgG4 reached a minimum level (3744.5±2690.9 vs. 4156.9±3344.5 mg, *P* = 0.33), the number of patients with elevated HbA1c after steroid therapy (one patient in the oral group had 1.0% elevation, and one in the pulse group had 0.9% elevation, *P* = 0.57) (Table 1). No other adverse events were seen.

**Table 1:** Background of patients^a^.

Items	Oral	Pulse	*P*
	(n = 8)	(n = 16)	value
Age (year)	56.0±9.4	60.0±9.6	0.41
Gender (men/women)	8/0	14/2	0.43
Type of pancreatic swelling (diffuse/partial)	3/5	5/11	0.55
Serum IgG4 before treatment (mg/dl)	367.5±287.2	476.0±409.3	0.27
Other organ involvement	4	9	0.56
Cholangitis	3	5	
Sialadenitis	1	1	
Retroperitoneal fibrosis	0	2	
Sialadenitis and retroperitoneal fibrosis	0	1	
Obstructive jaundice	4	8	0.67
PSL^b^ dosage until minimum serum IgG4 (mg)	3744.5±2690.9	4156.9±3344.5	0.33
Elevated HbA1c^c^ after steroid therapy	1	1	0.57

^a^Data are shown as a number or as the median±SD

^b^Prednisolone

^c^Glycosylated hemoglobin

**Fig 2:**
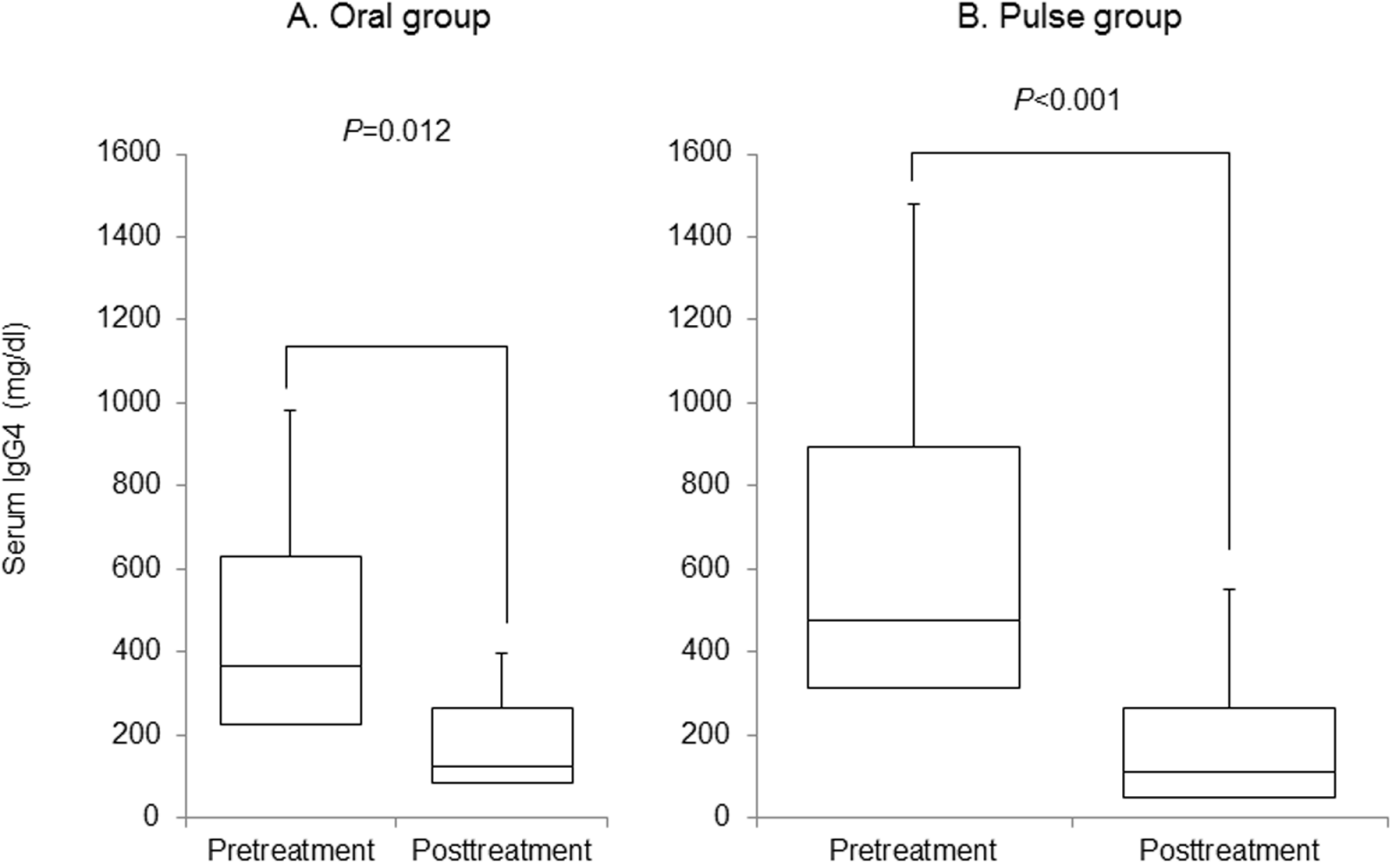
Serum IgG4 before and after steroid therapy. Serum IgG4 improved significantly in the oral group (A) and pulse group (B).

The serum IgG4 level was significantly reduced from 367.5±287.5 mg/dl before treatment to 124.3±129.3 mg/dl after treatment in the oral group (*p* = 0.012) (Fig 2A), and from 476.0±409.3 mg/dl before treatment to 110.3±147.6 mg/dl after treatment in the pulse group (p<0.001) (Fig 2B).

**Fig 3:**
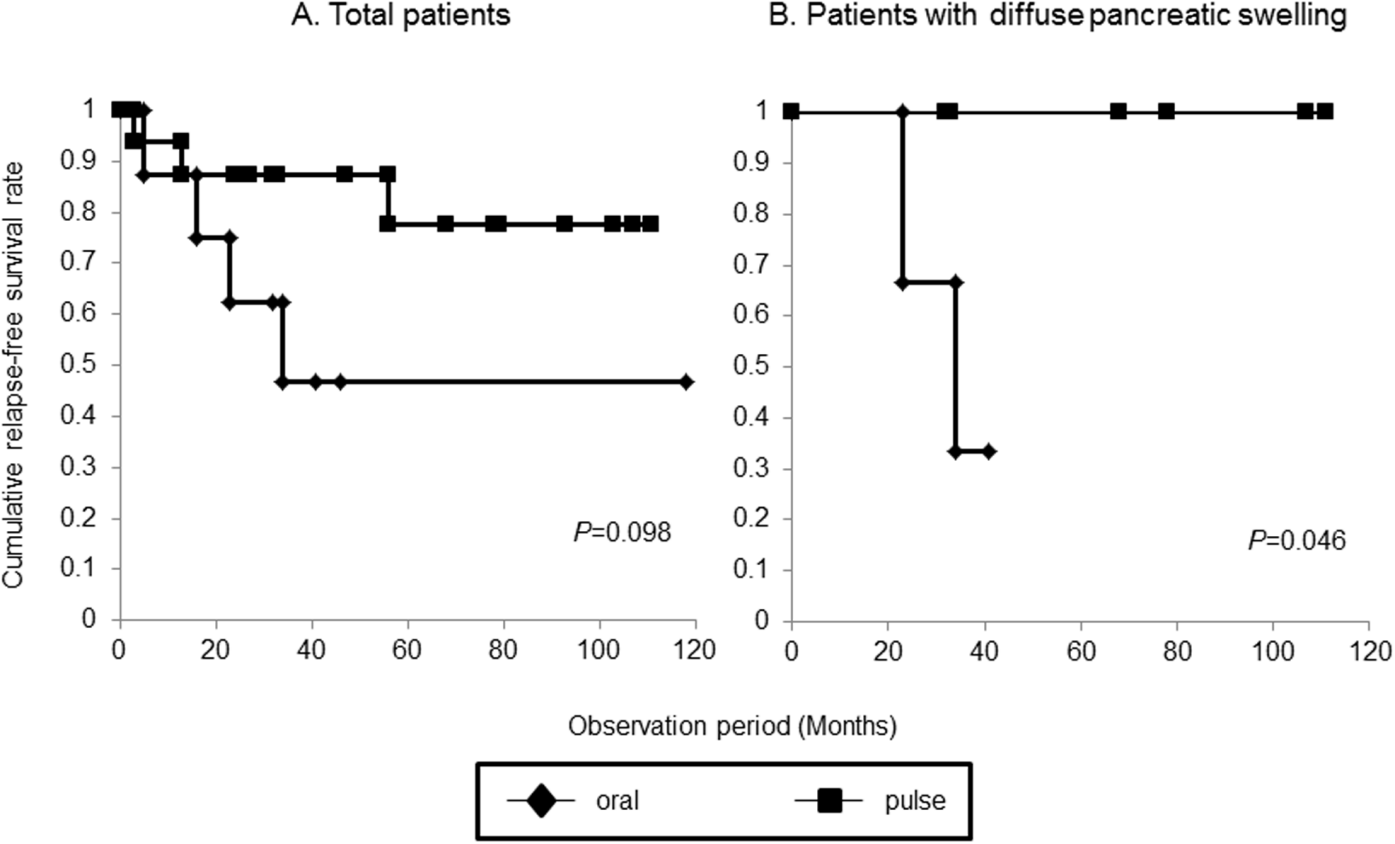
Cumulative relapse free survival rate. (A) There was no significant difference between the oral and pulse groups. (B) A significant difference was found in a comparison limited to patients with diffuse pancreatic swelling.

The 5-year cumulative relapse-free survival rate did not differ significantly between the oral and pulse groups (46.9% vs. 77.8%, *p* = 0.098) (Fig 3A). However, in a subset of cases with diffuse pancreatic swelling, this rate was significantly lower in the oral group (33.3% vs. 100.0%, *p* = 0.046) (Fig 3B)

## Discussion

In this study, the long term prognoses of patients who received oral steroid therapy or steroid pulse therapy were not significantly different, but a protective effect against relapse was found after steroid pulse therapy in patients with diffuse pancreatic swelling.

Steroid therapy has become standard therapy for AIP; however, regarding the time until relapse, a report from the Mayo Clinic found a 53% relapse rate after 3 months when steroid treatment was stopped at 11 weeks [13], whereas Kubota et al. found a relapse rate of 25.9% when steroid treatment was continued for ≥12 months [14]. Predictive factors for relapse of AIP include patient background factors and serum IgG4 levels. Patients with AIP in whom serum IgG4 did not elevate were excluded from the current study because these cases do not tend to relapse and can improve spontaneously [14]. In fact, the 5 cases in our hospital with serum IgG4 that was not elevated did not relapse. Among patient background factors, IgG4-related sclerosing cholangitis, obstructive jaundice, immune complex value, diffuse pancreatic ductal changes, and sclerosing sialadenitis have been proposed as relapse factors [6, 15–18]. Studies of the serum IgG4 level have proposed the following predictive factors for relapse: high serum IgG4 at initial diagnosis [11, 19–21], a high level before and after treatment [4], and resurgence of serum IgG4 in the remission phase after treatment [22].

In reports of IgG4-related conditions that were not limited to AIP, the serum IgG4 level was lowered by steroid treatment in all patients, but normalization was only achieved in about half of the patients, and patients with persistent high values were reported to be at risk for relapse [23]. The role of IgG4 in IgG4-related diseases is unclear, but Tabata et al. suggested that the serum IgG4 level reflects the disease activity of AIP [24]. In the current study, serum IgG4 improved in the oral and pulse groups, which suggests that the two treatments do not differ in their ability to reduce disease activity.

Steroid pulse therapy for AIP is useful for subsequent evaluation of the therapeutic effect due to the absence of a need for tapering [11] and may also improve liver function (ALT, γ-GTP) and HbA1c [12]. Steroid pulse therapy was found to be effective in a patient with AIP in whom lower bile duct stricture did not improve after oral steroid therapy, and improved γ-GTP on a long-term basis in this patient [12]. Our study indicated no significant difference in the effect on disease activity between steroid pulse therapy and oral steroid therapy. However, steroid pulse therapy was more effective than oral steroid therapy in patients with diffuse pancreatic swelling, and these patients may be suitable candidates for steroid pulse therapy.

The regimen of steroid pulse therapy in previous studies was mPSL 500 mg/day×3days. However, several adverse effects of this therapy have been reported: Zonana-Nacach et al. found a relationship between steroid pulse therapy (mPSL 1 g×3days) and dementia in a cohort study of 539 patients with SLE [25], and Haugeberg et al. showed that steroid pulse therapy (6.6–10 mg/kg) elevated the risk of osteoporosis [26]. There may also be an increased risk of failure of the circulatory system following administration of mPSL 500 mg within 10 minutes [27] and of liver dysfunction with steroid pulse therapy of mPSL at 500 mg/day [28]. For these reasons, as low a steroid dose as possible is preferable, and a regimen of steroid pulse therapy of (mPSL 250 mg or 125 mg/day) may be an option.

The limitations of the current study are the small number of patients and the steroid tapering dose not being perfectly in accord with the daily moving average, due to the retrospective nature of the study. However, the steroid dose tapering mostly used the methods referred to above and any deviation is likely to have had little influence on the results because all steroid doses during the period of serum IgG4 reduction did not differ significantly between the oral and pulse groups. As described above, steroid pulse therapy (mPSL 500 mg/day) is effective for therapeutic diagnosis of AIP [11]. We did not examine the effective dose of steroid pulse therapy (mPSL 125 or 250 mg/day) because all patients in the pulse group underwent steroid tapering. A future study on the effect of steroid pulse (mPSL 125 or 250 mg) without tapering is required to examine safer therapeutic diagnosis. However, further accumulation of patients is needed to confirm the results. Within these limitations, we conclude that steroid pulse therapy for AIP does not show a stronger effect than oral steroid therapy for prevention of relapse or on long-term prognosis, but pulse therapy may be an effective treatment for patients with diffuse swelling of the pancreas.
